# Identifying the individual metabolic abnormities from a systemic perspective using whole-body PET imaging

**DOI:** 10.1007/s00259-022-05832-7

**Published:** 2022-05-14

**Authors:** Tao Sun, Zhenguo Wang, Yaping Wu, Fengyun Gu, Xiaochen Li, Yan Bai, Chushu Shen, Zhanli Hu, Dong Liang, Xin Liu, Hairong Zheng, Yongfeng Yang, Georges El Fakhri, Yun Zhou, Meiyun Wang

**Affiliations:** 1 Paul C. Lauterbur Research Center for Biomedical Imaging, Shenzhen Institute of Advanced Technology, Chinese Academy of Sciences, Shenzhen, People’s Republic of China; 2 Department of Medical Imaging, Henan Provincial People’s Hospital and People’s Hospital of Zhengzhou University, Zhengzhou, People’s Republic of China; 3 Central Research Institute, United Imaging Healthcare Group Co., Ltd, Shanghai, People’s Republic of China; 4 Department of Statistics, School of Mathematical Sciences, University College Cork, Cork, Ireland; 5 Gordon Center for Medical Imaging, Department of Radiology, Massachusetts General Hospital, Harvard Medical School, Boston, MA USA; 6 School of Biomedical Engineering, Shanghai Tech University, Shanghai, People’s Republic of China

**Keywords:** Whole-body PET, Metabolic abnormality, Network analysis, Systemic disease

## Abstract

**Introduction:**

Distinct physiological states arise from complex interactions among the various organs present in the human body. PET is a non-invasive modality with numerous successful applications in oncology, neurology, and cardiology. However, while PET imaging has been applied extensively in detecting focal lesions or diseases, its potential in detecting systemic abnormalities is seldom explored, mostly because total-body imaging was not possible until recently.

**Methods:**

In this context, the present study proposes a framework capable of constructing an individual metabolic abnormality network using a subject’s whole-body ^18^F-FDG SUV image and a normal control database. The developed framework was evaluated in the patients with lung cancer, the one discharged after suffering from Covid-19 disease, and the one that had gastrointestinal bleeding with the underlying cause unknown.

**Results:**

The framework could successfully capture the deviation of these patients from healthy subjects at the level of both system and organ. The strength of the altered network edges revealed the abnormal metabolic connection between organs. The overall deviation of the network nodes was observed to be highly correlated to the organ SUV measures. Therefore, the molecular connectivity of glucose metabolism was characterized at a single subject level.

**Conclusion:**

The proposed framework represents a significant step toward the use of PET imaging for identifying metabolic dysfunction from a systemic perspective. A better understanding of the underlying biological mechanisms and the physiological interpretation of the interregional connections identified in the present study warrant further research.

## Introduction

Human metabolic homeostasis relies on complex neuronal, vascular, and humoral mechanisms at the level of the whole body. Simultaneous non-linear interactions among organs form distinct physiological networks. Many systemic diseases have an underlying disturbance in the inter-organ physiological interaction networks [1]. The existing methods for detecting such a disturbance work mostly at the organ level and developing generalized methodologies capable of adequately quantifying the abnormalities at the system level remain a challenge so far. Most of the studies conducted to date on such topics have utilized non-imaging tools. Thiele et al. [2] developed a metabolic network reconstruction approach in which organ-specific information from the literature and omics data were used. The data sources in that study included 20 organs, 6 sex organs, 6 blood cell types, and 13 biofluid compartments. Barajas-Martínez et al. [3] developed a physiological network based on anthropometric measurements, fasting blood tests, and other vital sign measurements. The authors concluded that the specific structural properties of the network change across the human lifespan and could, therefore, serve as indicators of the health status. Cui et al. [4] reconstructed the global mammal metabolic network for different tissues and cell types, through which they attempted to connect organs with the inter-organ metabolite transport. In separate studies, Bashan et al. [5] and Bartsch et al. [6] developed a framework to probe the interactions among diverse body systems and identified a physiological network that represented the interplay between network topology and function.

Imaging approaches have been used mostly to investigate the functional interactions related to brain dysfunction. Brain disorders, such as dementia, have their origin and the associated functional impairment not in distinct regions but rather in a network of connected regions [7]. Alterations in these inter-regional brain connectivity networks, if quantified, could reflect the status of various neurological diseases. For instance, structural connectivity has been investigated using diffusion tensor imaging (DTI) [8, 9], and functional connectivity has been investigated using functional magnetic resonance imaging (fMRI) [10–12]. DTI or fMRI connectivity patterns could, in principle, be determined on the subject level by correlating fiber connections or time-series signals. Metabolic connectivity deciphered using PET measurements, in contrast, is often derived using a population-based approach [13, 14]. The reason is that a routine PET scan usually performs static acquisition 60 min post injection, which measures the summed activity concentration in a certain period (typically 5–30 min). However, conventional methods cannot compute the connectivity (correlation) between the region without access to the real-time dynamic activity signal. Certain recent studies have suggested deriving the metabolic connectivity at the individual brain level based on relationships in the regional activities [15, 16]. On the other hand, few studies have investigated whole-body metabolic connectivity using PET imaging. Horsager et al. [17] used three different radiotracers to investigate the alpha-synuclein interaction pathology between organs, and the findings supported their hypothesis regarding the existence of two subtypes of Parkinson’s disease (brain-first top-down and body-first bottom-up types). Heiskanen et al. [21] utilized ^18^F-FDG PET to gain a system-level understanding of how exercise training affects the crosstalk in the central metabolism. Dias et al. [18] compared whole-body FDG uptakes and glucose metabolic rates between the diabetes group and non-diabetes group. They successfully derived difference of organ crosstalk in these two groups hence conclude the impact of diabetes on glucose homeostasis. Sundar et al. [20] compared the metabolic rates at multiple organs between healthy male group and female group, based on group-averaged normative correlation analysis of the measured time-activity curves. Suchacki et al. [19] reported an approach to understanding murine bone metabolic interactions in vivo by analyzing the correlations of the ^18^F-FDG time-activity curves in bones.

In this context, the present study proposes a framework capable of constructing a network that would reveal the individual metabolic abnormality using a subject’s whole-body SUV image and a normal control database. The analysis does not require access to dynamic acquisition, and is not limited to scanners with long axial field-of-view such as uEXPLORER  but can also be applied on conventional scanners. The key concept underlying the proposed framework is to model the individual differences based on the knowledge of normative modeling using a control database. The implementation of the proposed framework was demonstrated and validated in different diseases.

## Materials and methods

### Subject demographics

Twenty-four scans of healthy subjects with no diseases available in the records were used in the proposed framework. Twelve additional scans were used for testing, among which ten scans were those of a lung cancer diagnosis, one scan was performed 30 days after discharge upon recovering from the Covid-19 disease, and the remaining scan was for the case of gastrointestinal bleeding with the underlying cause unknown. The demographics of all subjects are presented in Table 1.

**Table 1:** Summary of the demographics of included subjects

Subject	Age (y/o)	Gender	Weight	Inject dose (MBq)
Total controls (***N*** = 60)	51.4 ± 13.9	28F, 32M	68.3 ± 12.2	259 ± 48
Controls for refNET (***N*** = 24)	50.4 ± 13.3	12F, 12M	69.7 ± 11.6	264 ± 51
Lung cancer (***N*** = 10)	55.5 ± 8.4	2F, 8M	74.4 ± 11.8	297 ± 71
Covid-19 discharge (***N*** = 1)	49	M	75	301
Gut bleeding (***N*** = 1)	49	M	65	265

### Data acquisition and processing

All scans were performed using a uEXPLORER PET/CT scanner (United Imaging Healthcare, Shanghai) in Henan Provincial People’s Hospital, China. The study protocols were approved by the local Ethics Committee. Written consent was obtained for each subject prior to scanning. The scan procedure and data formatting are as follows. First, a CT scan was performed for the attenuation correction. Next, a 60-min list-mode PET acquisition was initiated with the bolus injection of ^18^F-FDG into the vein in the lower extremity. In order to obtain the SUV image, the scan data for the 50–60 min time interval were reconstructed into a 192 × 192 × 80 matrix with a voxel size of 3.125 × 3.125 × 2.866 mm^3^ using the 3-D Ordered Subset Expectation–Maximization (OSEM) algorithm with time-of-flight information. The reconstruction was performed with 3 iterations, 28 subsets, and 2 mm Gaussian post-smoothing. Attenuation correction and scatter correction were performed using CT-based attenuation correction maps. Finally, the reconstructed activity image (in Bq/cc) was converted into an image with standard uptake values by normalizing it according to the injection dose and weight of the subject.

**Fig. 1:**
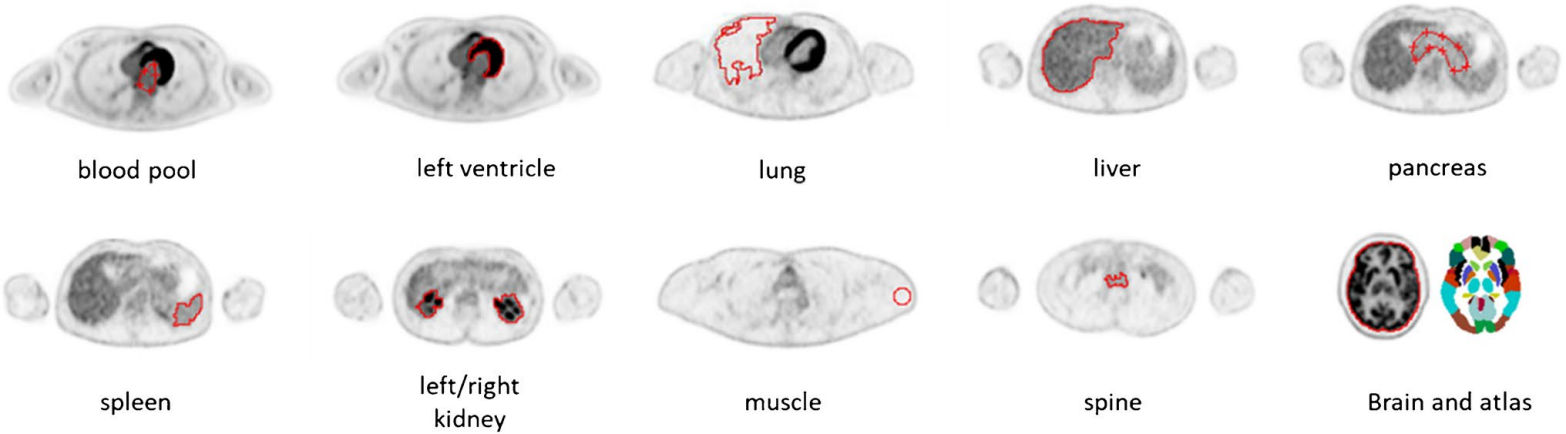
The delineation of the 18 sampled regions comprising 11 organs and 7 sub-regions of the brain. The left kidney and the right kidney were treated as two separate ROIs. The one for the lung was on the left or right where the lesion resides and excluding the lesion

For each scan, a total of 18 regions of interest (ROIs) were delineated on a given SUV image (Fig. 1). Among these ROIs, 11 were sampled in organs, including the whole brain, left ventricle, lung, liver, pancreas, spleen, left/right kidney, muscle, spine, and blood, while seven sub-brain regions were further parcellated using Statistical Parametric Mapping (SPM12) described as follows. The planes that contained the brain in the reconstructed image were extracted as a new volume and then spatially normalized according to an ^18^F-FDG PET template in the Montreal Neurological Institute (MNI) space. The normalized image was smoothed using a Gaussian filter with 8-mm FWHM and subsequently parcellated into 94 regions defined using the automated anatomical atlas (AAL2) [22]. In the present work, only brain stem, whole cerebellum (CER), cerebrospinal fluid (CSF), whole white matter (WM), caudate, putamen, and frontal cortex (SF) were selected for subsequent analysis. Next, network analysis was performed for the total 18 regions using the Brain Connectivity Toolbox [23]. The following statistical analyses were performed using the Statistics and Machine Learning Toolbox in Matlab R2018b.

### Network construction

**Fig. 2:**
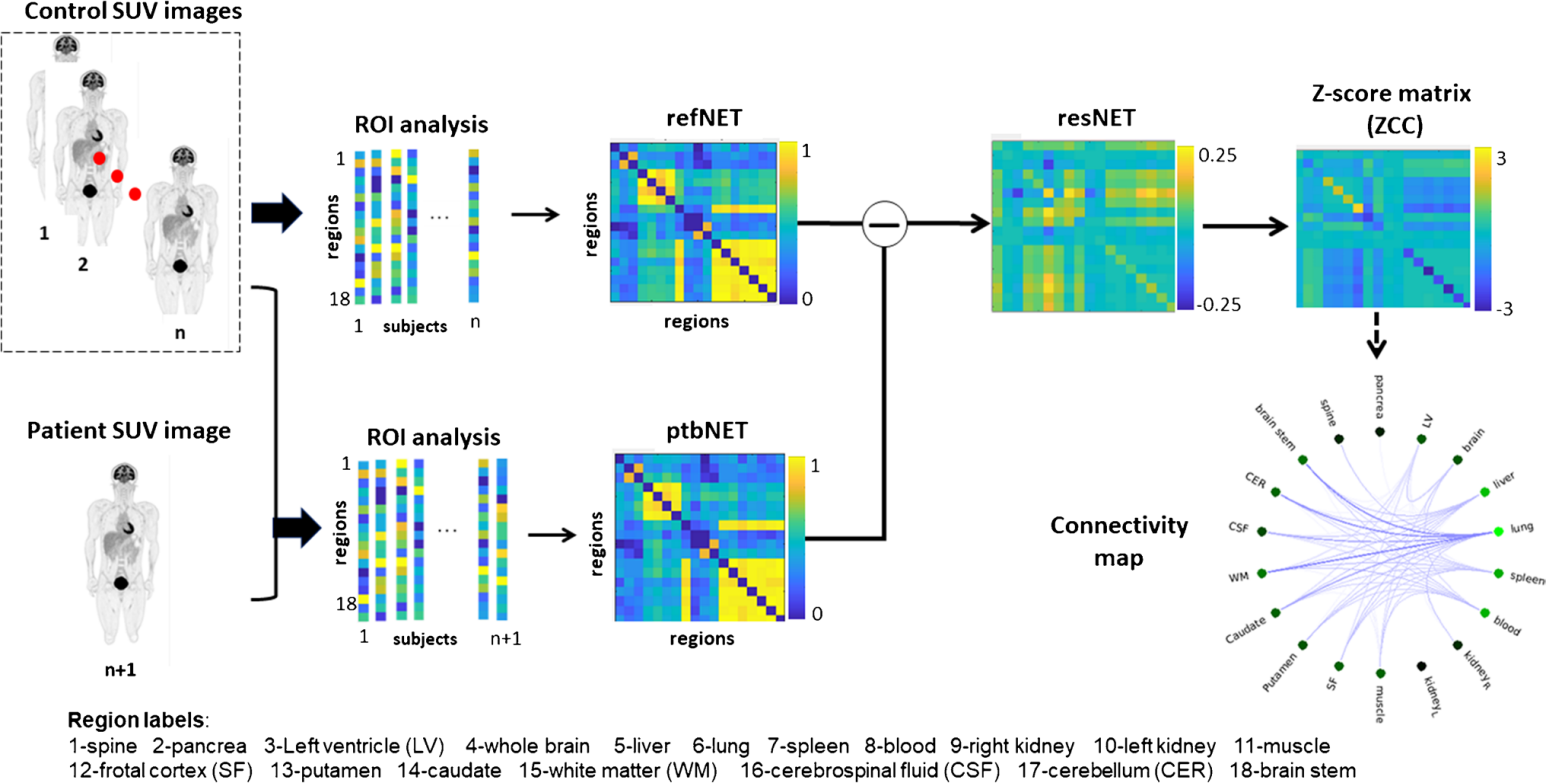
The proposed framework for obtaining the individual metabolic network from a patient scan. Reference network refNET is first constructed across all N controls, with each edge being the Pearson correlation coefficient between uptake values for each regional pair. Then, a new perturbed network ptbNET is constructed similarly by adding the patient to controls. The Z-score of the difference between the ptbNET and refNET can therefore be calculated as described by Eq. 1. The connectivity map can be plotted from the Z-score map for visualization

In order to construct the individual connectivity network using a single scan, we adapted a recently developed approach designed for anatomical brain MRI [24]. According to this approach, individual differences may be modeled based on the knowledge of normative modeling at the group level [25]. We adapted this concept to PET imaging as an overall framework illustrated in Fig. 2. First, a reference metabolic network **refNET** was constructed using the scans from the control group (24 healthy subjects) by performing the partial Pearson correlation analysis between the SUVs of each region pair, considering age and gender as covariates. The network matrix nodes represented the regions, while the edges represented the strength of the connection between the nodes, which was essentially Pearson correlation coefficient. The **refNET**, therefore, represented the common characteristics of all controls. Next, the subject was added to investigate the control group, thereby forming a new group with 25 subjects, which could again be constructed as a new structural covariance network matrix and was labeled as the perturbed network **ptbNET**. Further, the difference between the perturbed network **ptbNET** and the reference network **refNET** was calculated as the residual network **resNET**. A threshold of 30% was set to this network to eliminate the weak residual correlations after subtraction arising possibly due to noise. The Z-score map of **resNET** (ZCC) could then be calculated as follows:

1 $$\mathrm{ZCC }=\frac{\mathrm{resNET}-\mu }{\upsigma }=\frac{\mathrm{resNET}}{(1-{\mathrm{refNET}}^{2})/\left(\mathrm{N}-1\right)}.$$

Here, N denotes the total number of subjects in the new group, while $\mu$ and $\sigma$ are the mean and standard deviation of resNET. It was demonstrated that the Z-score map of resNET followed a symmetrical distribution, implying that µ equals zero [25]. The ZCC matrix essentially represented the level of abnormality in the connectivity of the 18 regions defined in Fig. 1. Each residual network and ZCC, therefore, contained 153 undirected edges connecting all regions (18 × (18 − 1)/2 = 153). Each edge exhibited a level of variation in metabolism, which led to deviation from the normal value in the control group. Under normal conditions, the body network structure is stable and relevantly linked. However, when the metabolism homeostasis is altered in a subject, the network links are altered in accordance. The connectivity map can then be plotted from the Z-score map for visualization as in Fig. 2. The degree of this alteration (abnormality) could be quantified by defining the strength of the abnormality (STR) at each node, as follows:

2 $${\mathrm{STR}}_{m}=\frac{\sum_{i\in \mathcal{M},i\ne m}{|\mathrm{ZCC}}_{mi}|}{\mathrm{M}-1}.$$

Here, *m* represents the region index, $\mathcal{M}$ is the set, and M denotes the number of regions. ${\mathrm{ZCC}}_{mi}$ is the correlation coefficient of the Z-score map between region *m* and its neighbor *i*. The total number of neighboring nodes equals M − 1.

### Data and statistical analysis

First, the overall consistency in the healthy control group was evaluated by measuring the within-group similarity, reproducibility, and the effect of the number of subjects. Next, the heterogeneity of the individual network in the patient group with lung cancer was determined. Subsequently, the measure from the individual-level network ZCC was compared to the one from the group-level analysis. Finally, the ability of the individual network to reveal the single-organ abnormality was evaluated by measuring the correlation between the SUV and the network strength STR defined in Eq. 2. The details regarding the implementation are provided below.

1. Control group reproducibility

First, the individual network analysis was performed for each control subject. The similarity among the control group subjects was measured by averaging the correlation coefficients of the Z-scores between any pair of networks. Next, a resampling procedure was performed to test the reproducibility of subject selection. The concept was that the refNETs associated with two randomly selected groups of control subjects should not present a statistical difference. Accordingly, 24 samples were selected randomly from 60 normal subjects as the control group, and this step was repeated 20 times. The refNET was then constructed from each randomly sampled group using the proposed framework. The reproducibility among these refNETs was quantified by averaging the correlation coefficients of the Z-scores between any pair of networks. Finally, the sensitivity analysis of the sample size was performed in the control group. The objective was to investigate the minimum number of subjects required to construct a control group. Accordingly, 10, 15, 18, 20, 28, 32, and 40 samples (20 times each) were selected from the 60 available normal subjects as the control group. The new associated resNETs were computed for each patient in the lung cancer group and then compared with the resNETs generated from the 24 subjects.

(2) Lung cancer group heterogeneity

Individual-level network analysis was performed for each lung cancer patient. The strength of each network was then compared to that of the reference network. The similarity among these patient networks was calculated by measuring the mean in-between subjects Pearson correlation coefficients for the pairwise Z-scores across all 153 edges.

(3) Group-level vs. individual-level network analysis

**Fig. 3:**
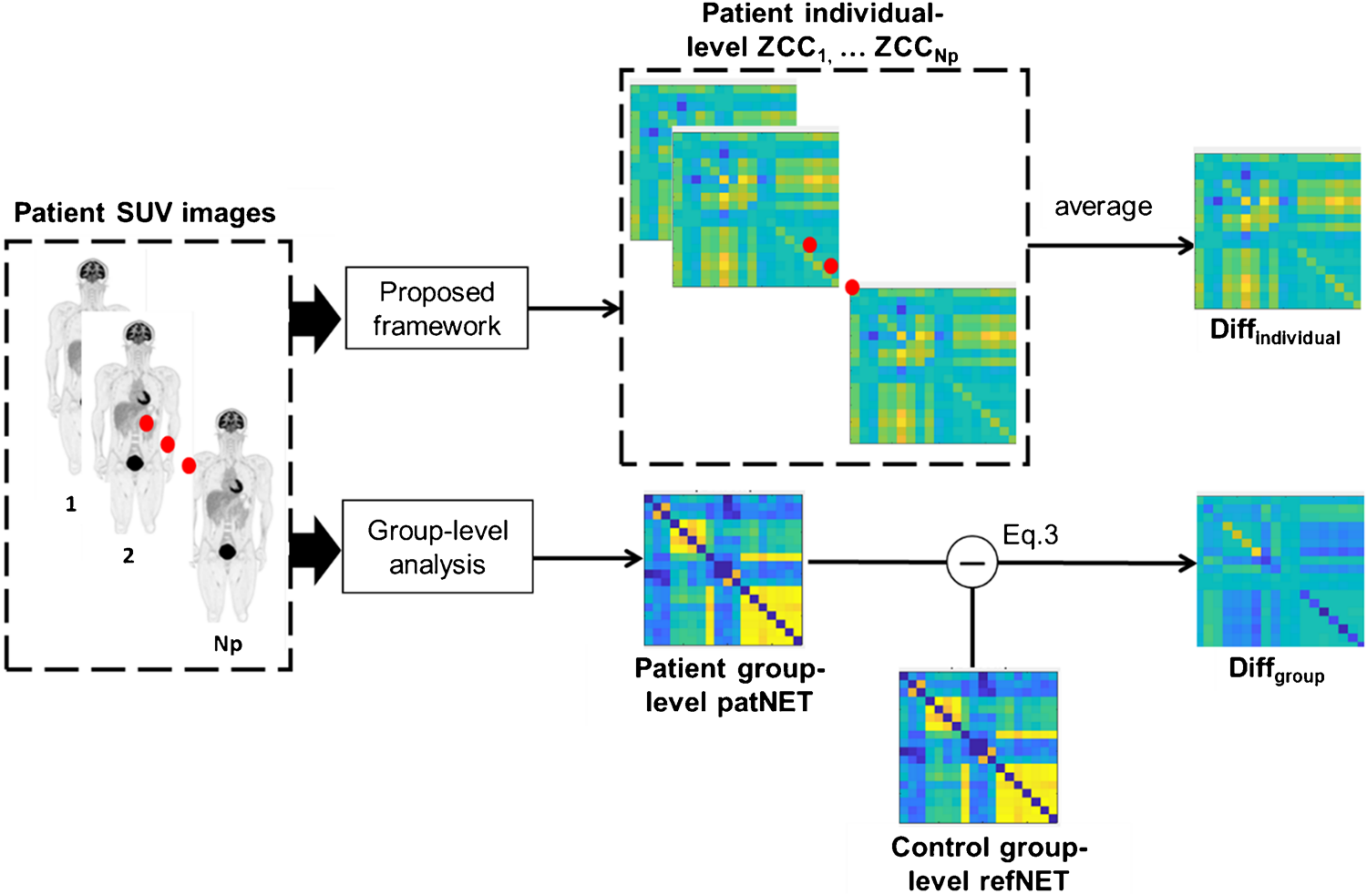
Illustration of the implementation of the group-level and individual-level analyses, and their comparison for the patient group with lung cancer

The proposed individual-level analysis was compared with the conventional group-level analysis, as shown in Fig. 3. The group-level metabolic networks were constructed for both patient and healthy control groups. Each network was constructed by calculating the Pearson correlation for the regional pairs for all subjects in that group. The normalized difference between the two group-level networks was considered the group-level difference network, defined as follows:

3 $${\mathrm{Diff}}_{\mathrm{group},\mathrm{i}}=\frac{{\mathrm{refNET}}_{\mathrm{i}}-{\mathrm{patNET}}_{\mathrm{i}}}{{\mathrm{refNET}}_{\mathrm{i}}+{\mathrm{patNET}}_{\mathrm{i}}}, i= 1\dots 153.$$

Here, the edge index is denoted by *i*, the patient’s group-level network is patNET, and the healthy control’s group-level network is refNET. Next, the mean of the Z-score at each edge across all patient resNETs generated using the proposed framework was calculated as the mean residual difference among the patients, as follows:

4 $${\mathrm{Diff}}_{\mathrm{individual},\mathrm{i}}=\frac{\sum_{j}{\mathrm{ZCC}}_{\mathrm{i},\mathrm{j}}}{{\mathrm{N}}_{\mathrm{p}}}, j\in {\mathrm{N}}_{\mathrm{p}}.$$

Here, *j* denotes the patient index and *N*_*p*_ is the total number of patients. The Pearson correlation coefficient between ${\mathrm{Diff}}_{\mathrm{individual},\mathrm{i}}$ and group-level difference network ${\mathrm{Diff}}_{\mathrm{group},\mathrm{i}}$ could then be computed.

(4) Individual network vs. single-organ analysis

The individual connectivity networks for one subject discharged after recovering from the Covid-19 disease and another subject with gastrointestinal bleeding were constructed. The change in the SUV was quantified as follows:

5 $${\Delta \mathrm{SUV}}_{\mathrm{m}}={\mathrm{SUV}}_{\mathrm{m}}\left(\mathrm{subject}\right)-\overline{{\mathrm{SUV} }_{\mathrm{m}}}\left(\mathrm{control}\right).$$

Here, ${\mathrm{SUV}}_{\mathrm{m}}(\mathrm{subject})$ denotes the uptake of the m-th organ for the subject to be investigated and $\overline{{\mathrm{SUV} }_{\mathrm{m}}}(\mathrm{control})$ denotes the average uptake of that organ in the control group. $\Delta \mathrm{SUV}$ was then compared against the network strength at the organ, defined in Eq. 2, to reveal the capability of the proposed method to detect the abnormality at the organ level.

## Results

### Control group homogeneity

Similarity within a group was measured by averaging the correlation coefficients of the Z-scores between any pair of networks. The similarity coefficient for the controls was determined to be 0.921 ± 0.133, which indicated a low inter-subject variability. The resampling procedure results also revealed a high level of reproducibility, and the average correlation coefficient between any pair of networks was 0.884 ± 0.141, which indicated the robustness of the method against the choice of control subjects. Further, the sensitivity analysis of the sample size in the control group was performed. The average similarity relative to 24 samples was determined to be 0.52, 0.58, 0.79, 0.848, 0.87, 0.878, and 0.871 for the sample sizes 10, 15, 18, 20, 28, 32, and 40, respectively, suggesting the robustness of the proposed network against the control sample size when the subject number was greater than 20.

### Lung cancer group heterogeneity

**Fig. 4:**
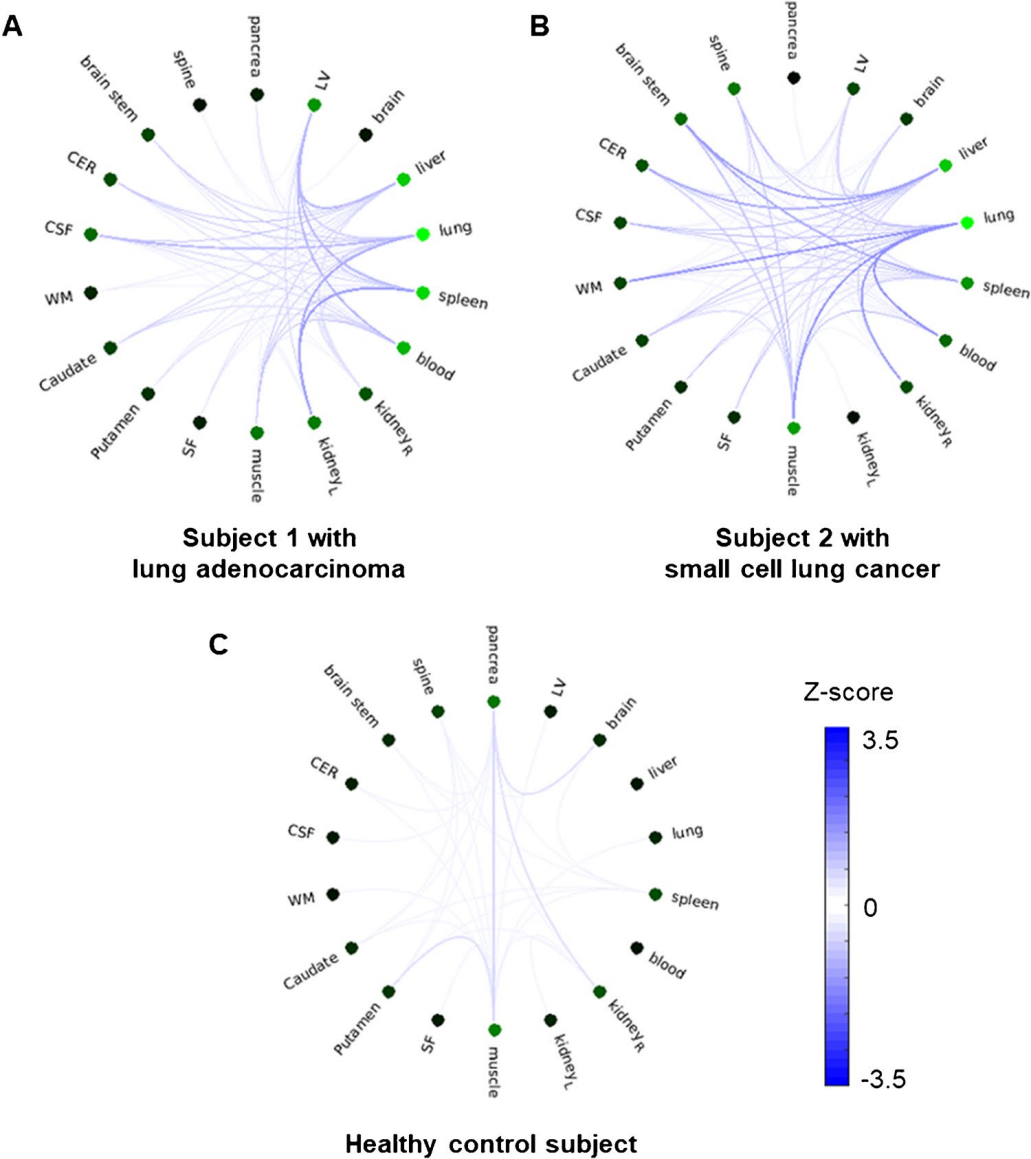
The connectivity plots for (**A**) and (**B**) patients with lung cancer and (**C**) a healthy control subject. The darker line indicates stronger edge connections between the nodes. The intensity of the green color indicates the strength at a particular node (dark green is stronger). Both connection degree and node strength exhibited differences between the patient and the control

**Fig. 5:**
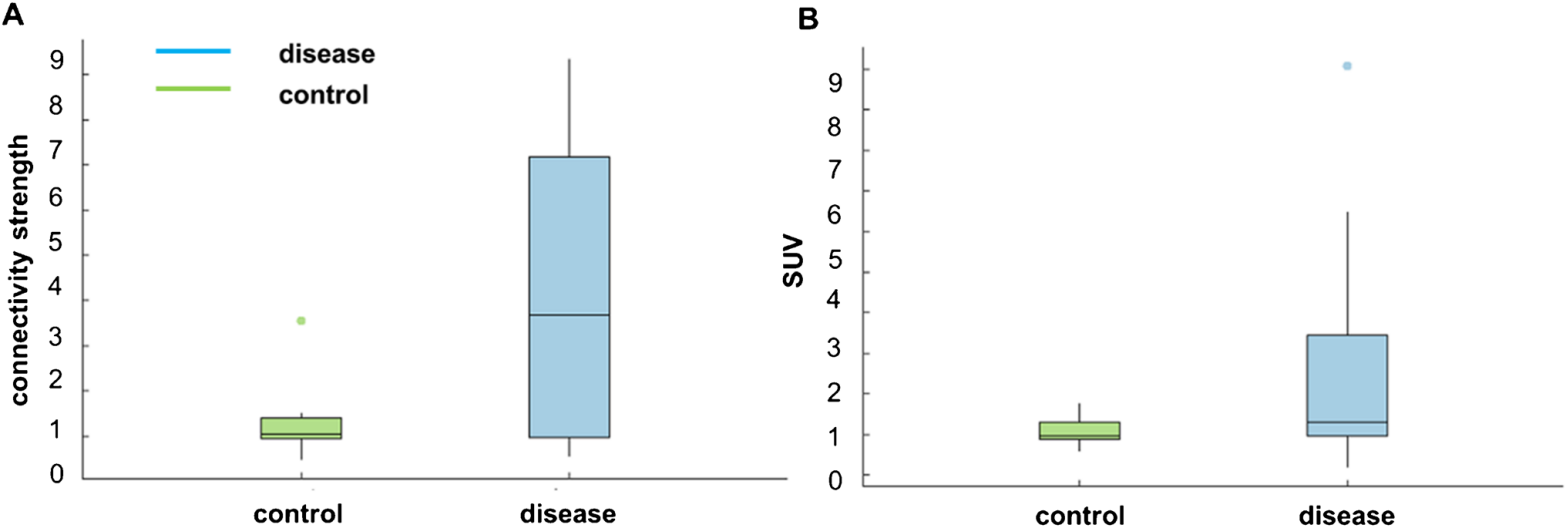
Summary boxplots of (**A**) the individual connectivity strength at lung for the control and disease groups and (**B**) the corresponding SUVs at lung (50–60 min). The connectivity strength of the individual network appears to be more capable of separating the control group from the disease group

The individual connectivity network for each lung cancer patient was investigated. An example of the individual-level network analysis is presented in Fig. 4. The strength of each patient’s individual network was significantly different from that of the reference network (*P* < 0.01; Bonferroni corrected for 153 edges). However, the similarity among the patient networks was low, with the mean in-between Pearson correlation coefficient among the Z-scores maps determined to be 0.196 ± 0.182. Despite the evident organ-wide heterogeneity across individual patients, the connections involving the lung exhibited higher abnormality compared to the other connections (Fig. 4). In order to further demonstrate the differences in the alteration degrees between the disease group and the control group, the summed strength at all nodes was computed for each individual network (Fig. 5). The average summed strength in the disease group was determined to be 6.3, while that in the control group was 2.1. This result indicated that the overall abnormalities or strengths of the altered edges might be more capable of separating the subjects with potential disease from the healthy ones.

### Individual-level vs. group-level network analysis

In order to understand the relationship between the proposed individual-level analysis and the conventional group-level analysis, group-level networks were constructed for both the control group and patient group. The group-level difference network ${\mathrm{Diff}}_{\mathrm{group}}$ and the mean individual-level network ${\mathrm{Diff}}_{\mathrm{individual}}$ were defined as illustrated in Fig. 3. The Pearson correlation coefficient between the mean individual-level difference network ${\mathrm{Diff}}_{\mathrm{individual}}$ and the group-level difference network ${\mathrm{Diff}}_{\mathrm{group}}$ was 0.78, which suggested that each subject contributed to the group-level difference. However, high heterogeneity existed among the subjects, as demonstrated by the results for the lung cancer group.

### Individual-level network vs. single-organ analysis

**Fig. 6:**
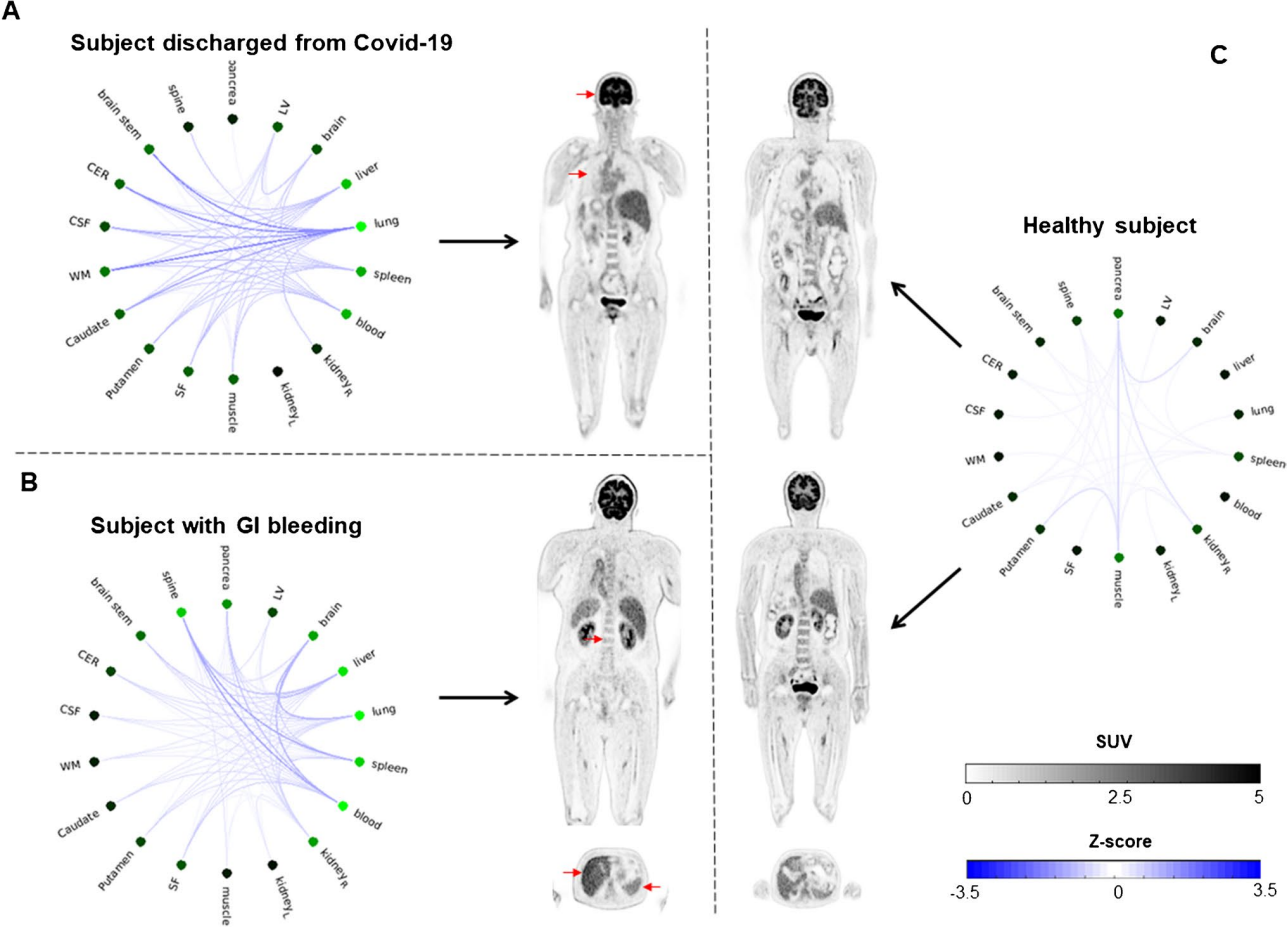
Metabolic connectivity plots for two abnormal subjects and a control. The images in the middle are the coronal SUV slices, and the plots on the two sides of these images are the network connections between the organs. In comparison to the control network, the networks of the abnormal subjects demonstrated denser connectivity and higher strength at the relevant nodes (corresponding to the abnormal uptake in the SUV image labelled with red arrows)

In the last analysis, individual connectivity networks were constructed for one subject discharged after recovering from the Covid-19 disease and one subject with gastrointestinal bleeding. As visible in Fig. 6, there was abnormal metabolic connectivity between the organs. In the case of the subject discharged after recovering from the Covid-19 disease, the lung, as an abnormal hub, exhibited the strongest connection strength, particularly with the brain (Fig. 6A). This may suggest this individual has triggered massive inflammatory response in multiple organs even after weeks of discharge. In the case of the subject with gastrointestinal bleeding, the blood, and the spleen functioned abnormally and had connections to the low uptake in the spine (Fig. 6B). An underlying abnormal condition related to hematopoiesis may be present for this individual, as it is known that both bone marrow and spleen contribute to this process.

**Fig. 7:**
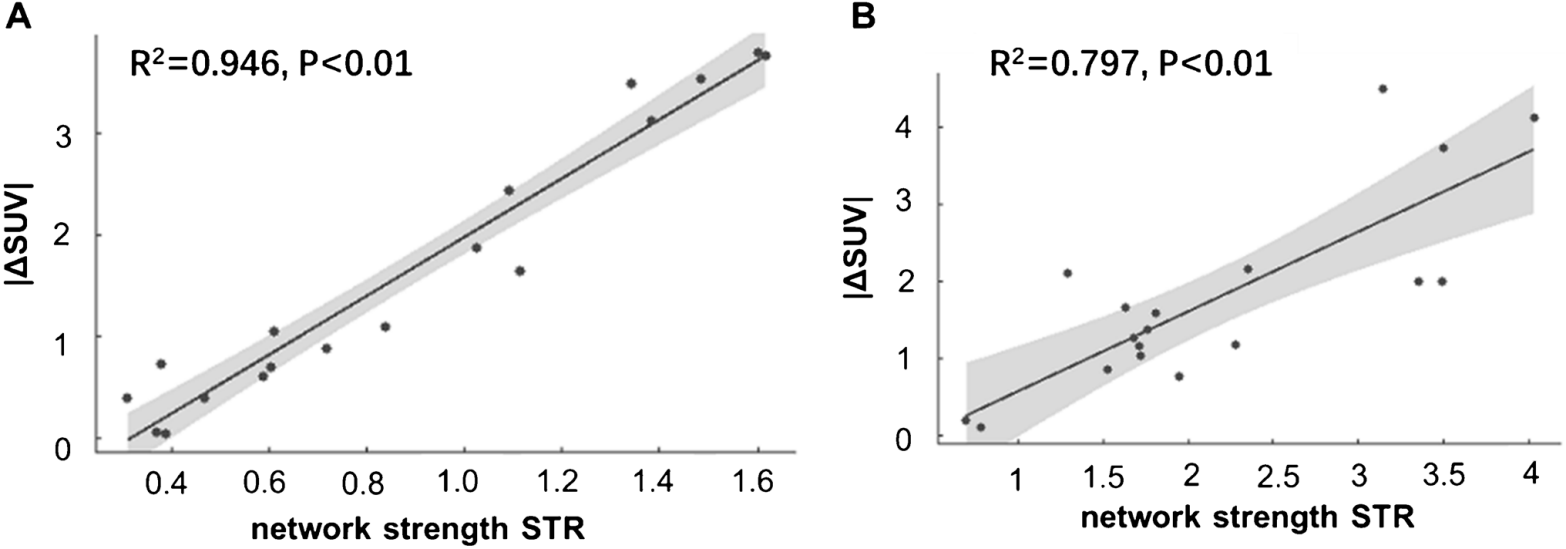
Correlation plots between the |ΔSUV| and the network strength presented in Fig. 6A,B at all sampled regions

Furthermore, node strength was significantly correlated (*R*^2^ = 0.946 and *R*^2^ = 0.797; *P* < 0.05) to |ΔSUV| at the organs (Fig. 7), which reflected the level of deviation of the SUV from the average value in the control group. This result indicated that an individual network could reveal the organ-level metabolic abnormality at a level comparable to the conventional uptake measures.

## Discussion

The present study proposed a framework that applies network principles for the analysis of whole-body PET data, thereby serving as a platform for identifying the metabolic dysfunctions at the system level, which is not possible to achieve using the existing analysis methods. It only requires whole-body SUV images that are either available from a multi-bed protocol on a scanner with regular axial FOV, or from a single-bed protocol on a long axial FOV scanner. The proposed framework is, in a way, complementary to the conventional methods from the network perspective. Examples of the application of this proposed framework include investigating the inter-functional crosstalk between the brain and other damaged organs in various diseases, such as visceral pain, or assessing the systemic metabolic change after chemotherapy or immune therapy. The conventional assessments, on the other hand, would focus solely on the focal response in such cases. Moreover, the proposed framework enables performing an individual-level analysis for identifying the potential systemic metabolic abnormality, which is not possible using the conventional group-level approaches due to the large heterogeneity across the disease group. This heterogeneity could be due to the differential disease expression or systemic functional alteration.

It is noteworthy that the proposed method does not provide a real metabolic connectivity network for a scan. What the network provided is a perturbation network against the reference, which reflects the variation between the normal samples and disease samples at the system level. Although a control group is essential for the analysis, the required number of healthy subject scans is not excessively large. Another potential approach to derive the metabolic network at the subject level would be utilizing dynamic PET. For example, the time–activity curves of sub-brain regions were successfully correlated at the subject level [19, 26, 27]. However, the limitation of this approach is that regional kinetics carry information of non-specific tracer binding and delivery, which could hide the tracer-specific interaction with its targets [28, 29].

The present study is a preliminary step that has introduced the concept of metabolic networks into the field of whole-body PET imaging. Therefore, certain limitations remain to be addressed. First, although it was confirmed that all the included scans were without visual artifacts, motion correction could nonetheless be required prior to network construction, although this requirement is often ignored in whole-body PET imaging [30]. Second, the delineation of the region sampled at each organ was performed manually, except for the brain. Ideally, the delineation should be performed automatically on the CT image, using a pre-trained neural network if possible [31]. Third, the data analysis to support the clinical diagnosis could be challenging because of the potential multiple pathologies present in a single patient. Similarly, low variability in the control group is a requirement when using the proposed framework. However, the physiological uptake difference is known to exist in healthy subjects. In addition, certain diseases or metabolic uptake abnormalities may remain undetected and are difficult to perceive in advance. This concern could be resolved by selecting a large number of control samples with distinct expression profiles, as this would increase the discriminatory power of the proposed framework [25]. Or at least, the distinct expression of the target disease in the controls has to be much less compared to that in the disease population, such as patients with lung cancer in the present study. Fourth, the connectivity pattern might be age dependent or sex dependent. Therefore, individual networks constructed for different age groups could provide further insight. Finally, the sensitivity of the individual network analysis to the scan protocol was not validated. Although the scan duration of all the scans included in the present study was the same, other scan/reconstruction parameters could vary across different centers and vendors. For example, conventional scanners perform the whole-body scan in multiple bed positions, which cannot guarantee all organs scanned at the same time window. Total-body scanners can relieve this problem as it can capture the uptakes of all organs at same time points.

Although in the present study, the individual metabolic network was constructed using ^18^F-FDG SUV images, this approach is adaptable to the application of other functional parameters, such as net metabolic rate, blood flow, and phosphorylation rate, when a dynamic ^18^F-FDG scan is available. Similarly, it may also be applied to non ^18^F-FDG tracers, such as those that visualize the neurotransmitters [32, 33], to reveal the brain–organ interaction dysfunction at the subject level. Nonetheless, the biological interpretation of the inter-regional connections, the dedicated applications using various radioligands, and the potential benefits of using which in personalized medicine warrant further research.

## Conclusion

The present study proposes a framework capable of constructing an individual metabolic abnormality network using a subject’s whole-body SUV PET image. The derivation of the scan from the normal group was achieved at both system and organ levels. Furthermore, using the proposed framework, the individual molecular connectivity of glucose metabolism was characterized. The proposed framework would serve in complementation to the conventional methods from the network perspective, thereby being potentially useful for systemic and other diseases. The complete potential of the proposed framework and the benefits of its application to specific clinical fields remain to be investigated in future studies.

## Abbreviations

PET: Positron emission tomography
SUV: Standardized uptake value
^18^F-FDG: 18F-Fluorodeoxyglucose
DTI: Diffusion tensor imaging
MRI: Magnetic resonance imaging
fMRI: Functional magnetic resonance imaging
OSEM: Ordered subset expectation–maximization
CT: Computerized tomography
ROIs: Region of interests
SPM: Statistical parametric mapping
MNI: Montreal Neurological Institute
FWHM: Full width half maximum
CER: Cerebellum
CSF: Cerebrospinal fluid
WM: White matter
AAL2: Automated anatomical atlas 2
